# Contagion of Consumption

**Published:** 1869-07-01

**Authors:** 


### *Contagion of Consumption.*

M. Chauveau, Professor in the Lyons Veterinary School, continues perseveringly his researches on the contagiosity of tuberculosis. He has of late selected the intestinal surface as the field for his investigations, and through it, by introducing tuberculous matter into the circulatory current, he has produced at will general tubercle. The *Union Médicale* reports that he lately purchased four handsome heifers, and he tuberculized three of them by causing them to swallow 30 grammes each of tuberculous matter taken from the body of an old phthisical cow. The rapidity of the result was extraordinary. At the end of twenty days the first heifer had lost flesh to a surprising extent, its pulse was quick and full, and it coughed incessantly. At the end of fifty-two days it was killed, and it presented perfectly marked tuberculous lesions, situated especially about the mesentery and intestine. The mesenteric glands presented infiltration in so high a degree, that many were of the size of the fist. Their total mass weighed 1,650 grammes. All the ganglia of the bronchi and mediastinum were enlarged, and the lung was full of crude tubercle. The other two heifers presented not less perfectly marked alterations, while the fourth, to whom none of the tuberculous matter had been administered, remained intact, and increased in flesh.

It is proved, therefore, that animals of the bovine species contract tuberculosis by digestive ingestion, just as they take carbuncle and cowpox, as sheep take the rot, as the horse takes glanders, and as man takes smallpox. The human digestive tube constitutes an easy channel for tuberculous contagion. If bovine phthisis be identical with tuberculosis in the human species, there is, in the use of the flesh of tuberculous animals, a danger to which the poor are more especially exposed.—*Medical Press and Circular, March 24.*

